# EHMTI-0212. Possible dependence of primary headache intensity on environmental factor as nuclear plant

**DOI:** 10.1186/1129-2377-15-S1-B13

**Published:** 2014-09-18

**Authors:** AS Harutyunyan, AY Avetisyan, HR Vekilyan, AH Karapetyan, EM Gevorgyan, HM Manvelyan

**Affiliations:** 1 Neurology, 2nd hospital, Yerevan, Armenia; 2 Neurology, Yerevan State Medical University, Yerevan, Armenia

## Introduction

Recently the incidence of primary headaches has been increased not only in the capital city of Yerevan, Armenia but in the rural distant towns too. There is consideration that in Armavir it could be associated with locally situated Nuclear Plant.

## Aims

The aim of this study was comparison of primary headaches in Yerevan and Armavir, and possible differences of disease distribution, headache pain intensity and impact of headache.

## Methods

100 patients with primary headaches were examined (50 in Yerevan and 50 in Armavir). Patients were also screened by visual analog scale (VAS) and headache impact test (HIT-6) screening tools.

## Results

Distribution of patients with tension-type, migraine and cluster headaches in Yerevan and Armavir was 82%, 11%, 7% vs 67%, 18%, 15%, respectively. Headache pain intensity by VAS was 5-7 in Yerevan compared with 7-8 in Armavir and HIT-6 score was 52 vs 58 in Armavir.

## Conclusions

Headaches distribution in Yerevan was within the international data, whether in Armavir the prevalence of paroxysmal types (migraine and cluster) is higher, with worse scores in both pain intensity and impact of headache.

No conflict of interest.

